# Microwave-Prepared Quantum Dots and Their Potential Applications as Adsorbents and Chemosensors

**DOI:** 10.3390/ma16206722

**Published:** 2023-10-17

**Authors:** Hebat-Allah S. Tohamy, Mohamed El-Sakhawy, El Barbary Hassan, Samir Kamel

**Affiliations:** 1 Cellulose and Paper Department, National Research Centre, 33 El Bohouth Str., Dokki, Giza 12622, Egypt; hebasarhan89@yahoo.com (H.-A.S.T.); elsakhawy@yahoo.com (M.E.-S.); 2 Department of Sustainable Bioproducts, Mississippi State University, P.O. Box 9820, Mississippi State, MS 39762, USA

**Keywords:** quantum dots, fluorescence, cellulose, chitosan, biochar, sensing, adsorption

## Abstract

A combination of different eco-friendly materials prepared promising fluorescent quantum dots (QDs) through the one-step process using the microwave heating of urea with cellulose, chitosan, and biochar. Characterizations of the prepared QDs, including the investigation of their structure by infrared spectroscopy, Raman analysis, X-ray diffraction, thermal gravimetric analysis, morphology, and optical properties, were performed. The results showed that QDs possess a small size, high UV absorption, and excitation wavelength-dependent fluorescence. The prepared QDs were also tested for metal ions removal from aqueous solutions. The adsorption at different contact times was investigated to optimize the adsorption efficiency of the prepared QDs. All QDs were found to be an ideal sorbent for Cr(II), Cu(II), Mn(II), and Pb(II). From the data, Cr(II) was more highly adsorbed than other metal ions. The results of the kinetic investigation showed that the pseudo-second-order kinetic model fit the adsorption process effectively. In addition, the fluorescence spectra of QDs were changed after the adsorption of metal ions; hence, the prepared QDs could be utilized in environmental sectors such as wastewater pollution detection, adsorption, and chemical sensing applications.

## 1. Introduction

Carbon quantum dots (CQDs) and graphene quantum dots (GQDs) are examples of carbon-based nanomaterials [1,2]. They have a large surface area and particle size of less than 10 nm [3]. GQDs are CQDs with zero-dimensional (i.e., dimensionless (0-D)) graphene (G) sheets. GQDs have functional groups incorporating oxygen (e.g., C-O-C, C=O, and OH). CQDs are nanoparticles with a ball form (NPs) made of diamond-like sp3 hybrid carbon and sp2 hybrid carbon sheets [1,2]. Several methods have been used to create CQDs, including heating organic molecules via hydrothermal and solvothermal processes, the laser ablation of graphite, and pyrolytic carbonization [1]. According to several researchers, microwave heating is suitable for developing more efficient and less expensive CQD synthesis methods. So far, it has been discovered that typical CQDs feature sp^2^ hybrid carbon cores and functional groups that contain O and N dispersed across their entire surface. A layer of hydrophilic groups surrounds the naturally hydrophobic carbon cores to protect them, creating a functionalized CQD dispersion that is biocompatible and stable in water [1,2,3]. Furthermore, CQDs are fluorescent dyes with distinct optical properties. CQDs have been utilized successfully in wastewater treatment due to their biocompatibility, low cost, and environmental friendliness [1].

Due to their sustainability, polysaccharides (e.g., cellulose and chitosan) are frequently used to create carbon materials [1]. On the other hand, a carbon-rich solid substance called biochar is created by pyrolyzing biomass waste. It could be used to treat water due to its remarkable ability to remove various pollutants from aqueous solutions. It can remove different heavy metal ions such as As (V) and As (III) [4], Ni, Pb, and Cu [5], and anions such as nitrate and phosphate ions [6]. Dyestuffs such as methyl orange [7], methylene blue [8] and other organic contaminants such as trichloroethylene from aqueous solutions can also be removed using biochar [9]. It is a cheap, renewable adsorbent that can be made using widely accessible biomaterials, making it appropriate for underdeveloped communities [10]. In addition, biochar preserves the organoleptic qualities of water [11].

The most up-to-date techniques for detecting hexavalent chromium in environmental samples rely on high-tech lab apparatus, like ion chromatography (IC) in conjunction with a guard column to eliminate of hydrophobic organics and an analytical column with a detection limit of 0.006 g/L. This method calls for extensive sample processing by trained scientists using pricey laboratory equipment after on-site sampling. Additionally, it has a limited throughput; sample processing costs money and takes a long time [12].

Many authors have recently considered treating wastewater by chemical, physical, and biological mechanisms to lessen its toxicity. These include biological treatments [13], electrocoagulation [14], aerobic biological treatments [15], and photocatalysis [16]. Adsorption is the most successful treatment for removing heavy metals among the available techniques since it may be handled without requiring higher temperatures, specialized techniques, or significant energy input [17,18,19].

Due to their advantages of extremely small size, optical properties, strong water solubility, customizable surface groups, low cost, and ease of fabrication, CQDs have received a great deal of attention in the disciplines of chemosensing [2,20,21,22,23]. In addition, the various active function groups on the surface make them a good candidate as adsorbents for different metal ions [1,2,3]. Tohamy et al. studied the effect of CQDs as adsorbents and chemo sensors for Cr(VI) and Pb(II). It was found that the removal efficiency was 83.85 and 96.48 for Cr(VI) and Pb(II), respectively [1,2]. In addition, the fluorescence quenching efficiency (FQE) was 49.57% for Cr(VI) [2]. Yao et al. studied the effect of a mixture of CQDs and QDs on the FEQ %, which was 60% [24].

Here, we present the synthesis of CQDs and GQDs from different sources using a microwave. Different techniques were used to confirm their structures. Their ability to adsorb Cr(II), Cu(II), Mn(II), and Pb(II) from aqueous solutions was studied with kinetic models. In addition, the fluorescence spectra were studied to investigate their suitability as chemosensors for different metal ions.

## 2. Materials and Methods

### 2.1. Materials

Dry bagasse was kindly provided by Quena Company of Paper Industry, Egypt, and ground to 450 μ. At Mississippi State University, rice straw was pyrolyzed at a feed rate of around 7 kg/h in a stainless-steel auger reactor to produce the biochar. The auger reactor was operated at a pyrolysis temperature of 425 °C, a residence time of about 1–2 s without a carrier gas or an added heat carrier, and nitrogen was utilized to exclude oxygen from the system [20]. Carbon, hydrogen, nitrogen content, oxygen (by subtraction), ash, and silica % were 41.47, 2.71, 0.80, 15.02, 40.1, and 28.9, respectively, which was determined using an elemental analyzer, the CE-440 (Exeter Analytical, North Chelmsford, MA, USA). Chitosan (medium molecular weight and deacetylation > 90%) was provided by Sigma-Aldrich (St. Louis, MO, USA). All of the chemicals were of the analytical grade and were utilized directly.

### 2.2. Methods

#### 2.2.1. Cellulose Extraction

Dry bagasse was hydrolyzed by HCl (1.5%) at 120 °C for 2 h with a liquid ratio of 1:10. With a liquor ratio of 1:7, sodium hydroxide (20%) was applied to the pre-hydrolyzed bagasse for 2 h at 170 °C. The lignin residue was removed by bleaching the treated bagasse with chlorous acid (HClO_2_). To get pure α-cellulose and eliminate any lignin remnants, the cellulose was mercerized using NaOH (17.5%) [25,26,27].

#### 2.2.2. Preparation of Quantum Dots (QDs)

Different mixtures of equal weights of cellulose, chitosan, and biochar were prepared using NaOH (0.21 g)/urea (7.2 g) system as follows:

(a) Cellulose, chitosan, and biochar.
(b) Cellulose and chitosan.
(c) Chitosan and biochar.
(d) Cellulose and biochar.

Each mixture was treated by domestic microwave at 700 W for 10 min, and the mixes turned into yellow/brown quantum dots and coded as C/CS/B QDs, C/CS QDs, CS/B QDs, and C/B QDs, respectively [1,3].

#### 2.2.3. Adsorption Study of Metal Ions

The prepared QDs efficiency for adsorption of Cr(VI), Cu(II), Mn(II), and Pb(II) from water was studied by adding each QD to individual metal solution at 25 ± 2 °C for 2 h, pH = 6.0 and shake at 200 rpm. HCl and NaOH were used to adjust the pH during the adsorption. The concentration of remaining metal ions was measured using PerkinElmer 3110, Waltham, MA, USA, spectrometer. The removal efficiency (*R* %) and adsorption capacities (qe) of CQDs were calculated using Equations (1) and (2):

(1) $$R\;\%=\frac{\left(C_{0}-C_{t}\right)}{C_{0}}\times 100$$

(2) $$q_{e}=\frac{\left(C_{0}-C_{t}\right)}{m}\times V$$

where *C*_0_ and *C_t_* are concentrations (mg/L) of metal ions before and after adsorption, respectively. *V* and *m* are the solution volume (L) and sorbent weight (g), respectively [1,3,25].

#### 2.2.4. Characterization and Analysis

##### Fluorescence Spectroscopy

Fluorescence microscopy was evaluated by the Spectrofluorometer model: Jasco FP-6500, Tokyo, Japan. Light source: Xenon arc lamp 150 Watt. Using the following formula, the fluorescence quenching efficiency (FQE) was determined:

(3) $$FQE=\frac{F_{0}–F}{F_{0}}$$

where *F*_0_ and *F* refer to the fluorescence intensity (F.I.) of the prepared quantum dots before and after adsorption of metal ions, respectively [2].

##### UV Spectroscopy

The UV–vis absorption spectrum was recorded by a UV–Vis spectrophotometer (JASCO V-630, Tokyo, Japan) using a 1 cm path length quartz cell. The quantum yield was calculated according to the formula:

(4) $$QY=Qst.\frac{mx}{mst.}\;{\left(\frac{ɳx}{ɳst.}\right)}^{2}$$

where “QY” is the quantum yield, “*m*” is the slope from the plot of fluorescence vs absorbance, “ɳ” is the refractive index of the solvent, the “*x*” indicates the unknown sample, and “*st*.” refers to methylene blue standard solution in water (0.1 M) [1,2].

##### Fourier-Transform Infrared Spectroscopy (FT-IR)

FTIR spectra were collected using the KBr disk method using a Mattson 5000 spectrometer (Unicam, Ilminster, UK).

##### X-ray Diffraction

The crystallinity was studied on X-ray powder diffraction as the diffraction patterns were measured by Bruker D-8 Advance X-ray diffractometer (Mannheim, Germany) applying a40 kV voltage and a 40 mA current employing copper (Kα) radiation (1.5406 Å).

CrI (%) = Sc/St × 100(5)

where Sc and St are the area of the crystalline and total domains, respectively [1,2].

##### Raman Analysis

Raman spectra were recorded at an excitation laser wavelength of 532 nm using Raman confocal WITEC Focus Innovations Alpha-300 microscope (Oxford Instruments, Abingdon, UK).

##### SEM/EDX

The SEM images were taken using Quanta/250-FEG (Thermo Fisher Scientific, Waltham, MA, USA) connected to an energy-dispersive X-ray analyzer unit adjusted at an acceleration voltage of 30 kV.

##### Thermogravimetric Analysis (TGA/DTG)

The sample was heated to 1000 °C at a rate of 10 °C/min under a N_2_ environment for the thermogravimetric analysis (TGA) of the produced quantum dots. To ascertain the activation energy (Ea) of the thermal deterioration, thermal analysis data were acquired. The Coats–Redfern method was used to apply Equations (6) and (7).

(6) $$log\;\left[\frac{1-{\left(1-\propto \right)}^{1-n}}{{T^{2}\left(1-\propto \right)}^{\;}}\right]=log\frac{AR}{\beta E}-\frac{E}{2.303\;RT}\;\;for\;n\neq 1$$

(7) $$log\;\left[\frac{-\log\left(1-\alpha \right)}{{T^{2}}^{\;}}\right]=log\frac{AR}{\beta E}\left[1-\frac{2RT}{E}\right]-\frac{E}{2.303\;RT}\;\;for\;n=1$$

where n, *α*, *β* (K/min), *T* (K), R (kJ/mol.K), A (s^−1^), and E are the order of degradation reaction, the fractional conversion, the heating rate, the temperature, the gas constant, the frequency factor and the activation energy, respectively. A straight-line correlation should be displayed when plotting a relationship using various suitable n numbers. As a result, the least square method was used by selecting several n values (ranging from 0 to 3.0), calculating the correlation coefficient (r) for each value of n, and estimating the standard error (SE). The frequency factor A was determined from the intercept (log AR/ßE) of the Coats–Redfern equation by the most suitable value of n, whilst the activation energy was calculated from the slope (E/2.303R). Equation (8) was used to calculate the other kinetic parameters, such as enthalpy (*H*), entropy (*S*), and free energy change (*G*) [3,26].

(8) $$∆H=E-RT;\;∆G=∆H-T∆S\;and\;∆s=2.303\;\left(log\frac{Ah}{kT}\right)R$$

where (*h*) and (*k*) are Planck and Boltzmann constants, respectively [25,26].

##### Kinetics and Isotherm Studies

This section applied kinetic models such as pseudo first-order and pseudo second-order to estimate the adsorption mechanism of different metal ions onto the prepared quantum dots. It can be determined from Equations (9) and (10).

Ln [q_e_ − q_t_] = ln q_e_ − K_1_t (9)

(10) $$\frac{t}{q_{t}}=\frac{1}{K2q_{e}2}-\frac{t}{q_{t}}$$

where *q_e_* and *q_t_* are the adsorbed amounts (mg/g) at equilibrium and time *t*, respectively. Ce is the adsorbate concentration after contact time *t*. K1 (min^−1^) and K2 (g/mg/min) are the pseudo first-order and pseudo second-order rate constants of adsorption. From the slope and intercept of the plot of t/qt against t, the values of *q_e_*2 and *K*2 were calculated, respectively [26].

The Weber–Morris intraparticle diffusion can be determined from Equation (11).

*q_t_ = k*_3_ *t*^1/2^ *+ c* (11)

where *k*_3_ and *C* are the intraparticle diffusion rate constant (mmol g^−1^ min^1/2^) and the slope that represents the thickness of the boundary layer [1].

The Langmuir and Freundlich isotherms could be determined from Equations (12) and (13), respectively [28].

(12) $$\frac{Ce}{q_{e}}=\frac{1}{Kq_{m}}-\frac{Ce}{q_{m}}$$

(13) $$\log q_{e}=\log Kf+\frac{1}{n}\log Ce\;$$

where *q_m_* (mg/g) is the maximum removal capacity and *Kf* is adsorption capacity.

Thermodynamic parameters could be investigated from Equations (14)–(16).

(14) $$\mathrm{ln Kd}=\frac{\Delta s}{R}+\frac{\Delta H}{RT}$$

(15) $$\mathrm{Kd}=\frac{Ci-Ce}{Ce}+\frac{V}{m}$$

(16) ΔG⁡−RTln⁡Kd

where Kd is the distribution coefficient on the surface of C/CS/B QDs, C/CS QDs, CS/B QDs, and C/B QDs. The values of ΔS and ΔH can be calculated from the intercept and slope by plotting lnKd versus 1/T [28].

##### Statistical Section

Every experiment was repeated three times, and the results were the average of the three. The results were drawn by OriginPro 2019b software, while Excel was used for statistical calculations.

## 3. Results

### 3.1. Caharacteizations

#### 3.1.1. FTIR Spectroscopy

**Figure 1:**
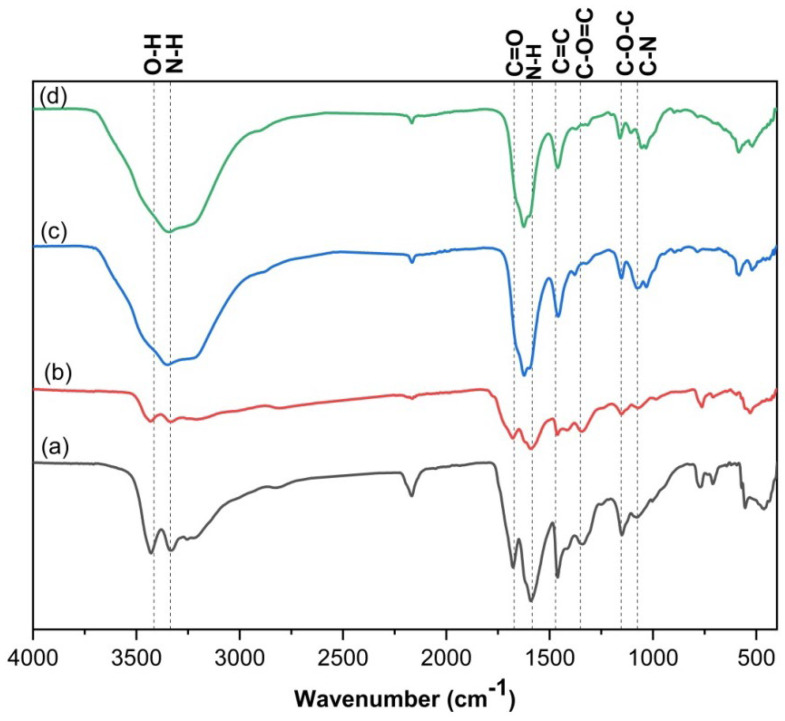
FTIR spectra of; (a) C/CS/B QDs, (b) C/CS QDs, (c) CS/B QDs, and (d) C/B QDs.

The IR spectra of the prepared QDs show absorption bands at 1625–1685 and 1693–1698 cm^−1^ assigned to the fingerprints of the amide II and I bands, respectively (Figure 1) [27]. A shift in the amide bands of the prepared QDs refers to the difference in the chemical structure of the starting materials. The bands at 3363–3429, 3241–3342, 1625–1685 and 1459–1463, 1346–1371 and 1151–1159, and 1062–1074 cm^−1^ are attributed to O–H, N–H, C=O, C=C, C–O=C, C–O–C and C–N stretching vibrations, respectively. In addition, the 1585–1598 cm^−1^ peak was attributed to N–H bending. N–H and C–N functional groups confirm nitrogen doping in the prepared quantum dots structures, which impart water stability during the adsorption process [1,2,3]. Also, the presence of O–H and N–H bonds improves the stability and hydrophilicity of QDs in an aqueous solution.

#### 3.1.2. Raman Analysis

**Figure 2:**
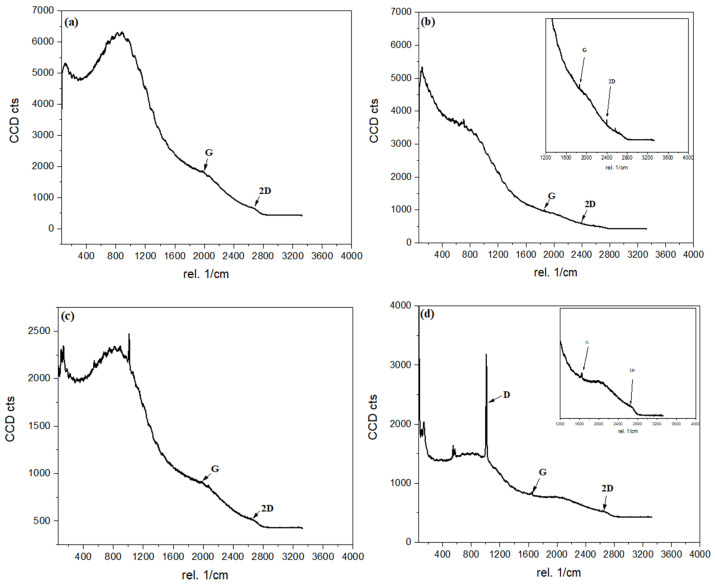
Raman spectra of; (**a**) C/CS/B QDs, (**b**) C/CS QDs, (**c**) CS/B QDs, and (**d**) C/B QDs.

Figure 2 and Table 1 compare the Raman spectra of C/CS/B QDs, C/CS QDs, CS/B QDs, and C/B QDs. The presence of a G peak is attributed to COOH functional groups. D peak is inactive for C/CS/B QDs, C/CS QDs, and CS/B QDs. As zigzag and armchair edge states emerge, the momentum conservation law gives rise to the D peak. It has been noted that the nature of the edge states in the graphene quantum dots is highly sensitive to the intensity of the Raman D peak. For the C/B QDs, the ID/IG (intensity of the D to G band) ratio is 2.02. For the armchair edges it is very present, however for the zigzag edge it is almost completely absent. So, C/B QDs has armchair edges, while C/CS/B QDs and C/CS QDs have zigzag edges [29]. At the same time, C/B QDs is amorphous compared to other QDs. This may be due to the formation of a large number of CQDs compared to others that contain graphene oxide. The sp^2^ sites were converted into sp^3^ sites by urea treatment. We might therefore say that the sp^2^ configuration is transformed from rings to chains in some sites due to nitrogen incorporation. This can be proved by the highest value of I_D_/I_G_ for C/B QDs compared to other quantum dots. This investigation is also confirmed by the highest N content as in the EDX analysis of C/B QDs, which indicated the deformation due to nitrogenization [30].

#### 3.1.3. X-ray Diffraction Study

**Figure 3:**
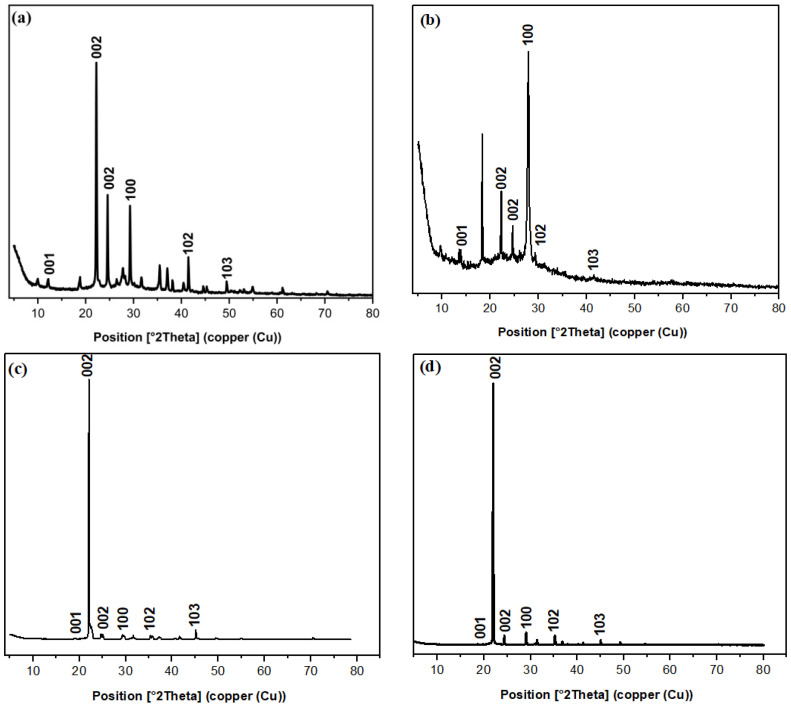
XRD pattern of; (**a**) C/CS/B QDs, (**b**) C/CS QDs, (**c**) CS/B QDs, and (**d**) C/B QDs.

The crystal structure of the quantum dots was confirmed by the XRD pattern (Figure 3). The XRD pattern showed the GO peaks known to exist, peaking at 2θ = 18.83, 18.33, 19.89, and 18.63° refer to the (001) plane, with the d spacing of 0.72, 0.64, 0.44, and 0.47 nm and at 2θ = 22.22, 22.24, 22.12, and 21.95° refer to (002) plane due to the presence of GO for C/CS/B QDs, C/CS QDs, CS/B QDs, and C/B QDs, respectively [1,2]. In C/CS/B QDs, increasing the d value is referred to as introducing more O- and N-containing groups. The XRD spectrum in Figure 3 confirms that the synthesized C/CS/B QDs, C/CS QDs, CS/B QDs, and C/B QDs are crystalline. The peaks at 24.57, 29.27, 41.48, and 49.47° for C/CS/B QDs; 24.64, 27.90, 29.31, and 41.50° for C/CS QDs; 22.62, 29.51, 35.75, and 41.62° for CS/B QDs and 24.43, 29.06, 35.22, and 45.05° for C/B QDs correspond to the (002), (100), (102), and (103) crystal planes in which (002), (100), and (102) represent graphite (sp^2^) and (103) represents diamond (sp^3^)-like carbon [1]. Respectively, the CrI % was 46.38, 47.02, 91.28, and 87.07% for C/CS/B QDs, C/CS QDs, CS/B QDs, and C/B QDs.

#### 3.1.4. Morphological Analysis

**Figure 4:**
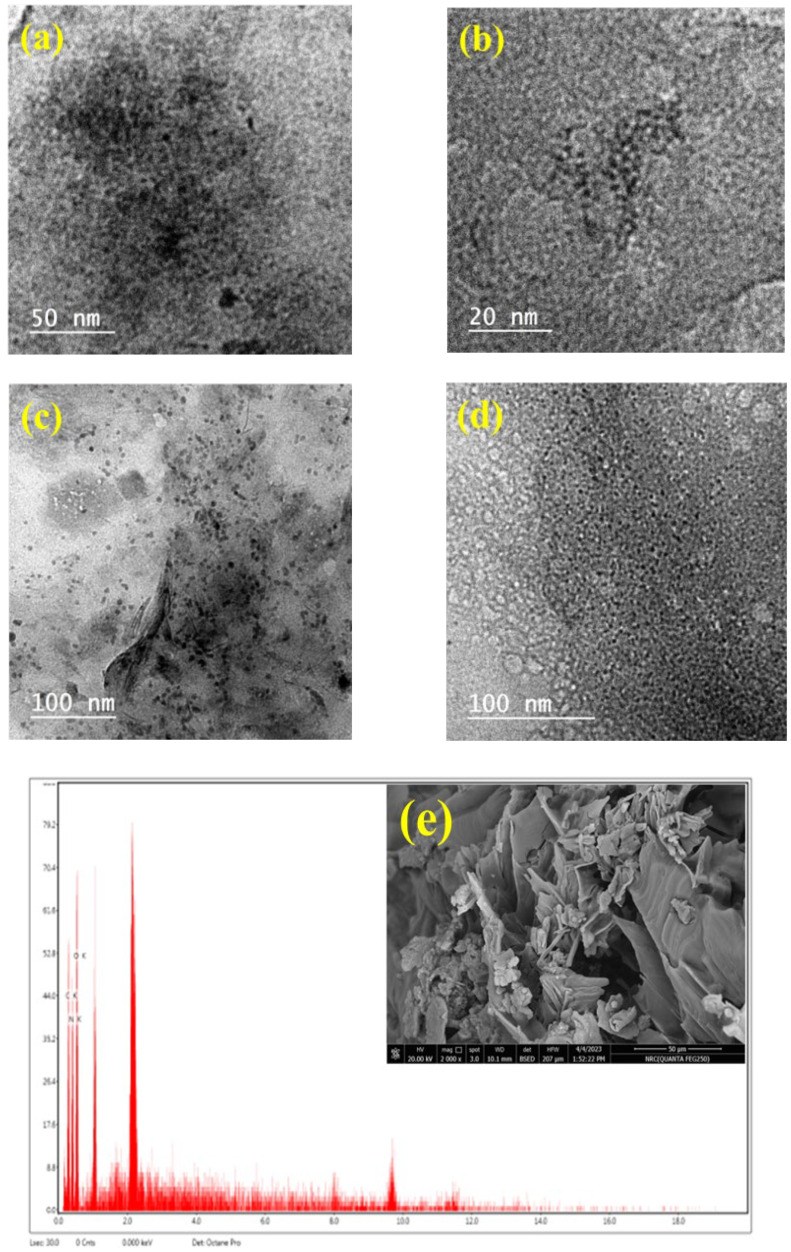

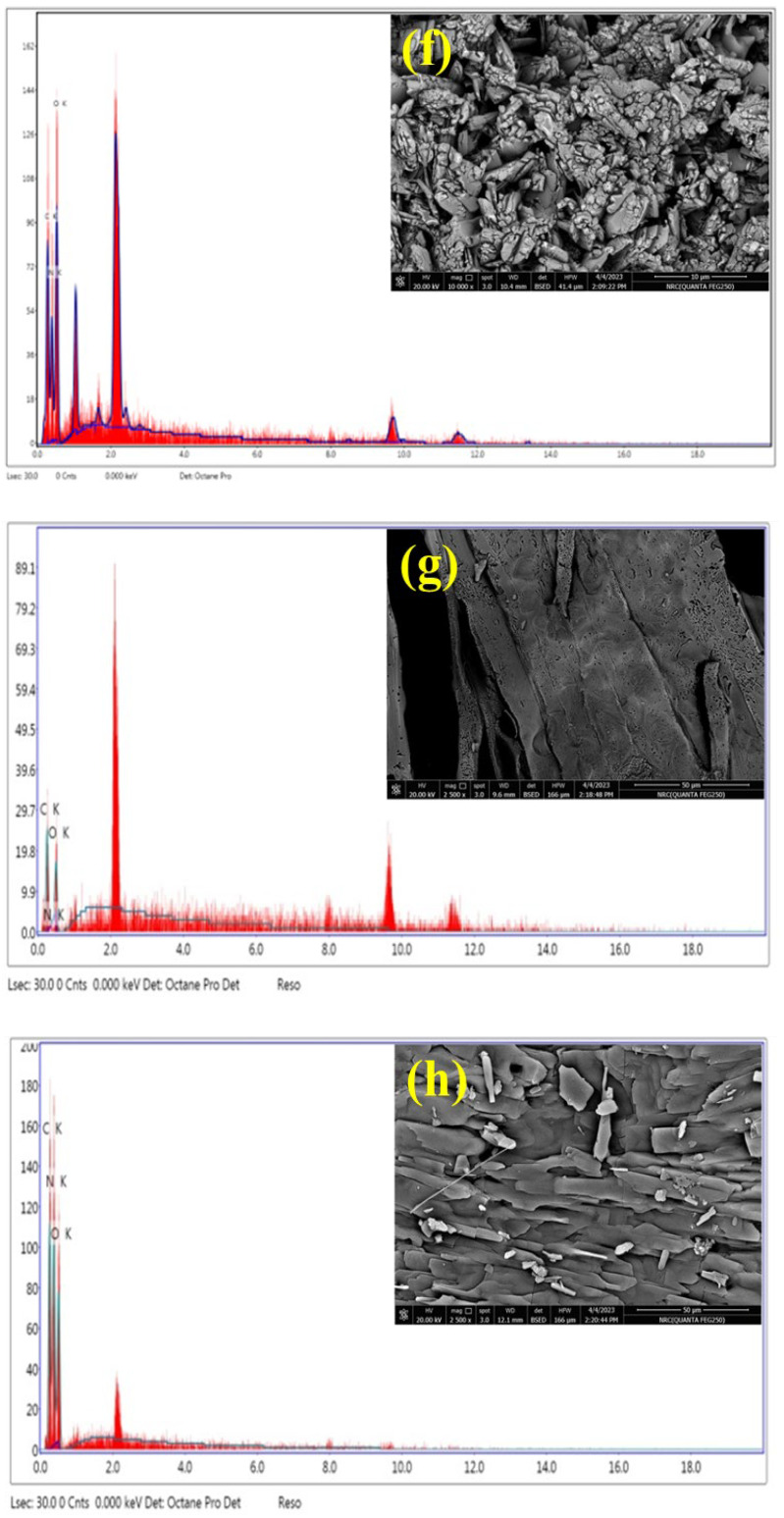
TEM (**a**,**b**,**c**,**d**) and SEM images with EDX analysis (**e**,**f**,**g**,**h**) of C/CS/B QDs, C/CS QDs, CS/B QDs, and C/B QDs, respectively.

TEM analysis of C/CS QDs revealed pure CQDs, while C/CS/B QDs, CS/B QDs, and C/B QDs revealed GQDs which are graphene sheets incorporated with CQDs (Figure 4). The appearance of fluffy sheets indicated the presence of graphene while pure, rounded structures indicated mainly to the CQDs. These investigations revealed that the presence of cellulose with CS makes pure CQDs (i.e., C/CS QDs), while the presence of cellulose with CS and/or biochar yield GQDs (i.e., C/CS/B QDs, and C/B QDs). In addition, mixing CS with biochar yields GQDs (i.e., CS/B QDs).

No obvious CQDs or graphene sheets appeared in SEM images except for C/CS/B QDs. This may be due to the crumbling of samples by storage. The degrees of nitrogenization (DN) calculated from EDX were 35.51, 33.31, 8.66, and 45.88%, C/CS/B QDs, C/CS QDs, CS/B QDs, and C/B QDs, respectively.

#### 3.1.5. Thermal Study

**Figure 5:**
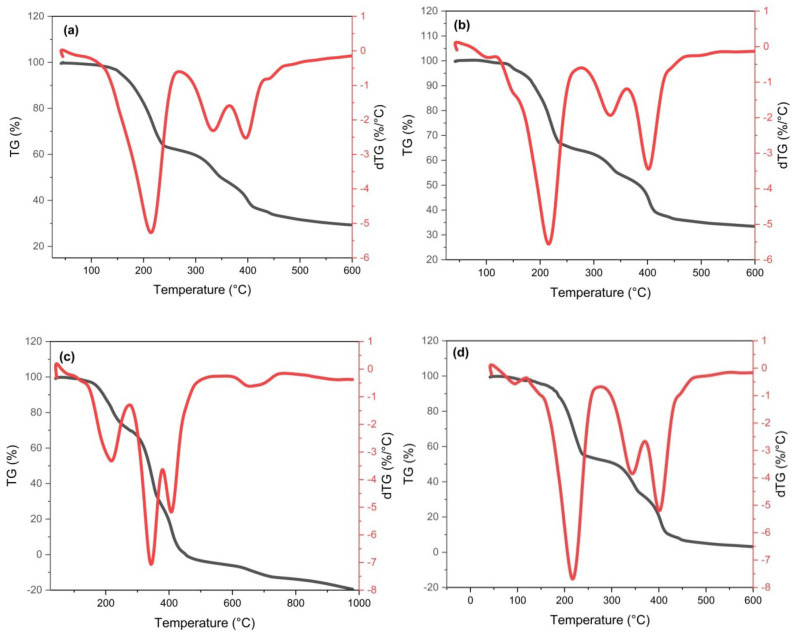
TGA/DTG of; (**a**) C/CS/B QDs, (**b**) C/CS QDs, (**c**) CS/B QDs, and (**d**) C/B QDs.

The prepared QDs were submitted to TGA under N_2_ to evaluate their relative long-term stability and segmental mobility at different times. Figure 5 displays the TGA traces and their derivatives (DTG curves), whereas Table 2 summarizes the information collected through their analysis and the kinetics of the decomposition processes. Generally, it can be observed that the CS/B QDs, and C/B QDs displayed similar behavior (i.e., Ea = 89.24 and 89.64 kJ·mol^−1^, respectively), this may be due to the higher biochar content that led to a higher stability and lower segmental mobility for CS/B QDs and C/B QDs. This result is consistent with XRD Cr.I (%). The TGA/DTG of C/CS/B QDs, C/CS QDs, CS/B QDs, and C/B QDs revealed residual weights (RW) of 23.34, 26.83, 58.50, and 48.88%, respectively, which suggested that some non-volatile components were present [26].

The C/CS/B QDs and C/CS QDs decomposition curves revealed two decomposition steps (Figure 5a,b). The first weight loss, due to the loss of moisture content, occurred between 41.82–264.60, and 41.70–276.06 °C, with a maximum of 214.05, and 215.76 °C, and mass loss (ML%) of 37.51, and 35.80%, respectively. The second main decomposition step was split between 266.27–357.27 and 357.40–978.72, and 277.83–361.05 and 362.71–979.81 °C, with a maximum of 331.02 and 397.27, and 330.05 and 400.36 °C, respectively, and ML% of 13.29 and 25.86, and 10.78 and 26.59%, respectively, was due to depolymerization and the combustion process [3]. The Ea for the first split step (i.e., 19.49 and 19.34 kJ mol^−1^) was higher than the second split step for the second decomposition step (i.e., 18.00 and 18.47 kJ mol^−1^), so it is suggested that the first split step is related to carbon core burn of CQDs (ML≈13.29 and 1.78%) [31]. At the same time, the CS/B QDs decomposition curves revealed three decomposition steps. The first weight loss occurred between 42.15–270.61 °C, with a maximum of 216.53 °C, and ML of 14.76%, which was due to the loss of moisture content. The second split main decomposition step was between 279.27–379.82, and 379.90–452.90 °C, with a maximum of 342.99, and 403.60 °C, respectively; and ML 18.56, and 8.18%, ascribed to the depolymerizations of organic matter (Figure 5c). The third decomposition step was between 598.23–759.90 °C, with a maximum of 663.30 °C and ML 13.16%, ascribed to the combustion process [2,3].

The prepared C/B QDs slightly showed the same decomposition steps. The first weight loss occurred between 45.26–119.92 °C, with a maximum of 96.38 °C, and ML of 2.55%, which was due to the loss of moisture. The second decomposition step was between 151.98–269.50 °C, with a maximum of 217.19 °C, ML 32.58% (Figure 5d). The third step was split between 282.05–371.05 and 372.16–979.23 °C, with a maximum of 340.47 and 402.43 °C, and ML of 6.18 and 9.8%, respectively. The Ea for the second decomposition step (i.e., 48.71 kJ mol^−1^) was higher than the first split step for the third decomposition step (i.e., 22.21 kJ mol^−1^), so it is suggested that the second step is related to carbon core burn of CQDs (ML ≈ 32.58%) while the first split step for the third decomposition step is due to depolymerization (i.e., Ea = 22.21 kJ mol^−1^). The second split step for the 3rd decomposition step is due to the combustion process [1,3].

From the previous results, we can say that the CS/B QDs and C/B QDs have a higher total RW (58.50 and 48.88%) compared to C/CS/B QDs (23.34%) and C/CS QDs (26.83%), which suggests that the CS/B QDs and C/B QDs need more temperature to degrade. The CS/B QDs and C/B QDs are more thermally stable due to the high content of biochar. Consequently, it needs a high temperature to deteriorate. According to Table 2, the values of ∆S are negative, indicating that the system degradation is non-spontaneous [3,26].

#### 3.1.6. Fluorescence Microscopy

**Figure 6:**
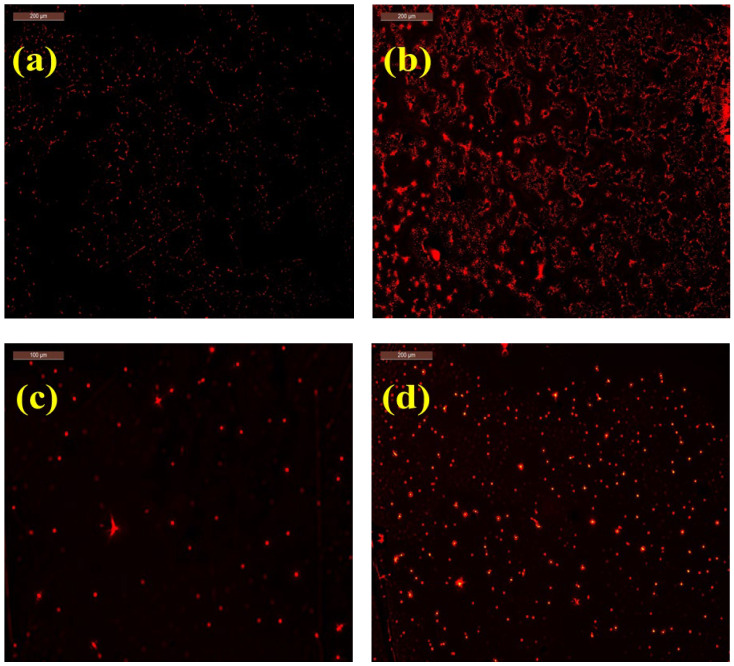
Fluorescence images of; (**a**) C/CS/B QDs, (**b**) C/CS QDs, (**c**) CS/B QDs, and (**d**) C/B QDs.

The fluorescence of prepared quantum dots was observed using a fluorescence microscope and the red fluorescence was observed in all prepared quantum dots with different intensities. Figure 6 shows the fluorescence images and the enrichment of CQDs around nucleoli, where the nucleoli became brighter and clearer in the case of C/CS QDs. This may be due to the creation of pure carbon quantum dots. On the contrary, the nucleoli became faint with weak contrast and smaller in the case of C/CS/B QDs, CS/B QDs, and C/B QDs. This may be due to the formation of GQDs consistent with the previous section’s TEM analysis.

### 3.2. Adsorption Study

The contact time effect on the adsorption efficiency of the prepared QDs for Cr(VI), Cu(II), Mn(II), and Pb(II) was studied at different times, namely 15, 30, 45, 60, 90, 120, 240, and 360 min. The affinity of C/CS/B QDs, C/CS QDs, CS/B QDs, and C/B QDs toward Cr(VI), Cu(II), Mn(II), and Pb(II) was not the same. In general, it was discovered that removal began quickly since there were more free functional groups and slowed with longer adsorption times. The Cr(VI) was more highly adsorbed due to its being defective by six electrons, while other metal ions, Pb(II), Cu(II), Cr(VI), and Mn(II), are made defective by two electrons only. Consequently, Cr(VI) has a faster chance of reducing to Cr(III) and forming a complex with BQDs that is rich in electrons [32].

**Figure 7:**
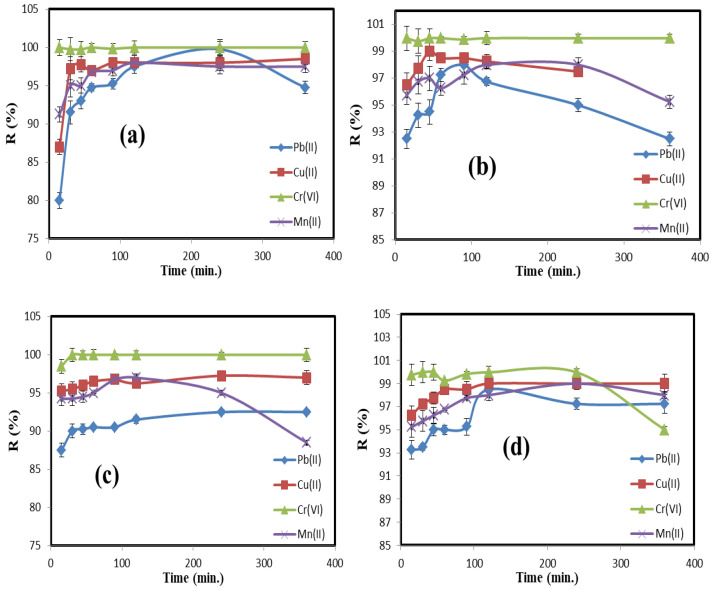

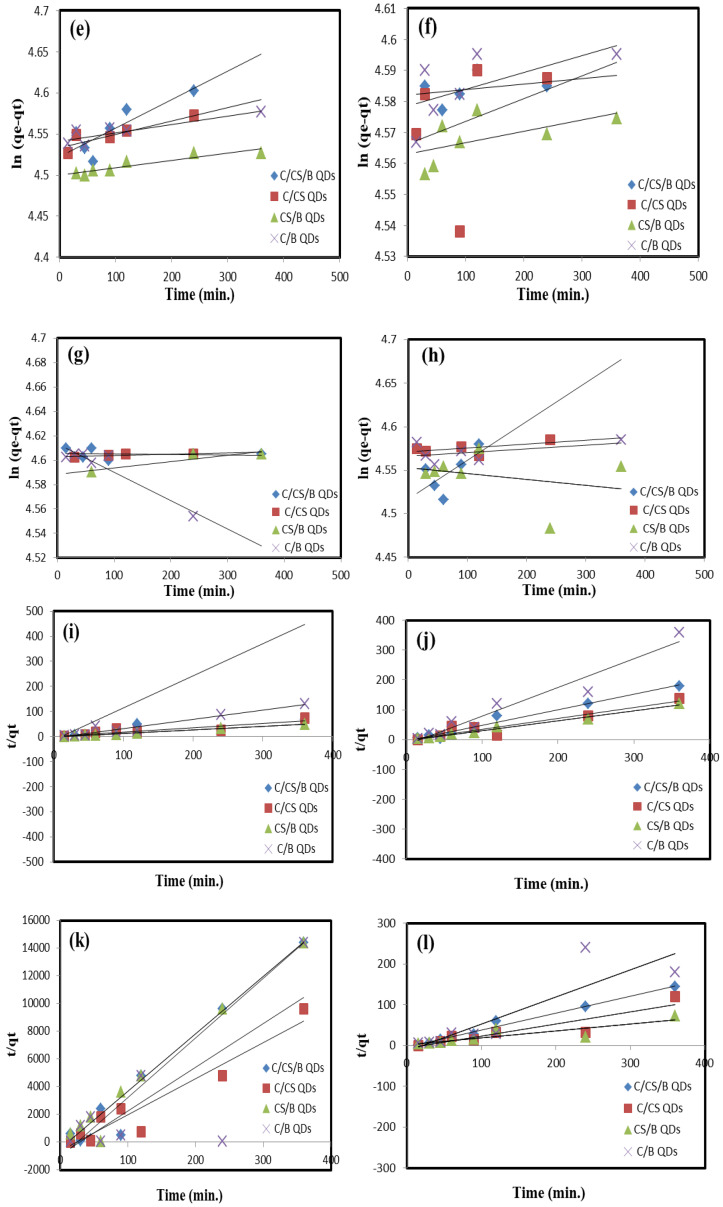

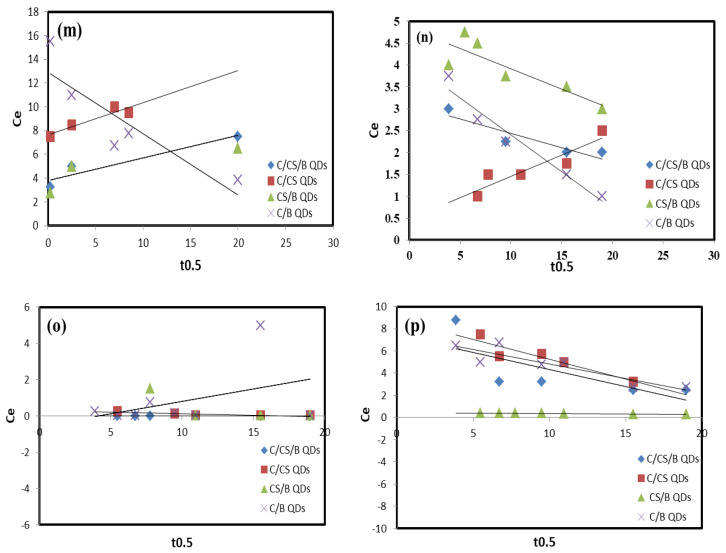
Effect of contact time on the adsorption efficiency of; (**a**) C/CS/B QDs, (**b**) C/CS QDs, (**c**) CS/B QDs, and (**d**) C/B QDs; with pseudo first-order rate for (**e**) Pb(II), (**f**) Cu(II), (**g**) Cr(VI) and (**h**) Mn(II); pseudo second-order rate for (**i**) Pb(II), (**j**) Cu(II), (**k**) Cr(VI) and (**l**) Mn(II); and intra-particle diffusion for (**m**) Pb(II), (**n**) Cu(II), (**o**) Cr(VI), and (**p**) Mn(II).

As shown in Figure 7a, there was no remarkable increase in the adsorption rate of C/CS/B QDs observed after 240, 360, 120, and 120 min for Pb(II), Cu(II), Cr(VI), and Mn(II), respectively. The R (%) of C/CS/B QDs towards Pb(II), Cu(II), Cr(VI), and Mn(II) was 99.75, 98.50, 99.97, and 98.00%, respectively. At the same time, there was no remarkable increase in the adsorption rate of C/CS QDs observed after 90, 60, 15, and 120 min for Pb(II), Cu(II), Cr(VI), and Mn(II), respectively. The R (%) of C/CS QDs towards Pb(II), Cu(II), Cr(VI), and Mn(II) was 98.00, 99.00, 99.97, and 98.00%, respectively. In addition, there was no remarkable increase in the adsorption rate of CS/B QDs observed after 360, 240, 30, and 120 min for Pb(II), Cu(II), Cr(VI), and Mn(II), respectively. The R (%) of CS/B QDs towards Pb(II), Cu(II), Cr(VI), and Mn(II) was 92.50, 97.25, 99.97, and 97.00%, respectively. Moreover, there was no remarkable increase in the adsorption rate of C/B QDs observed after 120, 120, 30, and 240 min for Pb(II), Cu(II), Cr(VI), and Mn(II), respectively. The R (%) of C/B QDs towards Pb(II), Cu(II), Cr(VI), and Mn(II) was 98.50, 99.00, 99.97, and 99.00%, respectively.

Figure 7e–g shows the effect of the sorbent type (i.e., pure CQDs or GQDs) on the removal efficiency of Pb(II), Cu(II), Cr(VI), and Mn(II). It was observed that Pb(II) removal was the highest in the case of C/CS/B QDs (i.e., 99.75%), Cu(II) removal was the highest in the case of C/B QDs (i.e., 99.00%), Cr(VI) removal was the same for all quantum dots ~99.97 and Mn(II) removal was the highest in the case of C/CS/B QDs, C/CS QDs, and C/B QDs (i.e., 98.00%).

The adsorption kinetics will be implicated in the Pb(II), Cu(II), Cr(VI), and Mn(II) adsorption. The pseudo first-order and pseudo second-order equations are utilized to model the kinetics of Pb(II), Cu(II), Cr(VI), and Mn(II) on C/CS/B QDs, C/CS QDs, CS/B QDs, and C/B QDs. For Pb(II) and Cu(II), concerning the values of qCalc as presented in Table 2, it is seen that the pseudo first-order model better fits the adsorption data for C/CS/B QDs, C/CS QDs, CS/B QDs, and C/B QDs, which means the bonds in the adsorption are chemical. At the same time, the R^2^ values obtained in the pseudo second-order are still suitable for describing the kinetics of Pb(II) and Cu(II) sorption. These values elucidate the surface processes involving chemisorption and physisorption in the adsorption of Pb(II) and Cu(II) by C/CS/B QDs, C/CS QDs, CS/B QDs, and C/B QDs [1,26].

For Cr(VI) and Mn(II), concerning the values of qCalc as presented in Table 2, it is seen that the pseudo first-order model better fits the adsorption data for C/CS/B QDs, C/CS QDs, CS/B QDs, and C/B QDs, which means the bonds in the adsorption are chemical. At the same time, the R^2^ values obtained in the pseudo second-order are still suitable for describing C/CS/B QDs, C/CS QDs, and CS/B QDs. These values elucidate the surface processes, involving chemisorption and physisorption in the adsorption of Cr(VI) by C/CS/B QDs, C/CS QDs, and CS/B QDs (Table 3). In contrast, C/B QDs are pure pseudo first-order (i.e., qCalc and R^2^), which means only chemisorption [26].

The intra-particle diffusion plots are shown in Figure 7g, observing that straight lines do not pass through the origin point. This behavior confirms that there are two stages of adsorption, i.e., surface adsorption of Pb(II), Cu(II), Cr(VI), and Mn(II) and intra-particle diffusion, which can be controlled by the surface adsorption of Cr(VI) onto C/CS/B QDs, C/CS QDs, CS/B QDs, and C/B QDs. This finding was attributed to the strong electrostatic attraction of the Pb(II), Cu(II), Cr(VI), and Mn(II) to the C/CS/B QDs, C/CS QDs, CS/B QDs, and C/B QDs surfaces, followed by the diffusion of Pb(II), Cu(II), Cr(VI), and Mn(II) into C/CS/B QDs, C/CS QDs, CS/B QDs, and C/B QDs pores [2].

**Figure 8:**
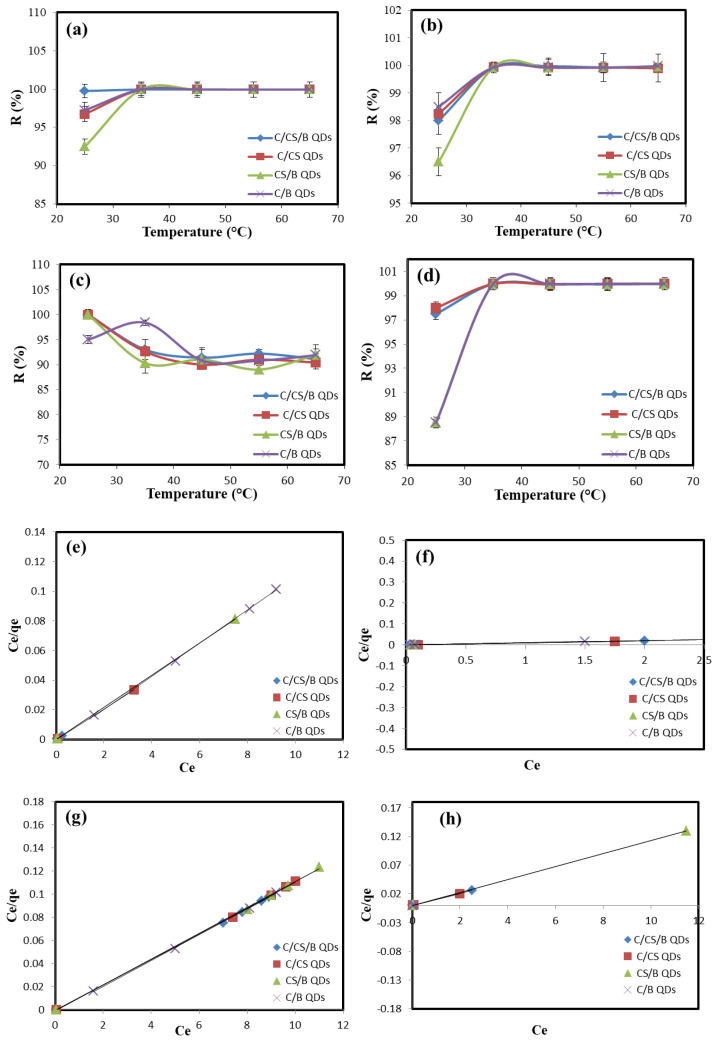

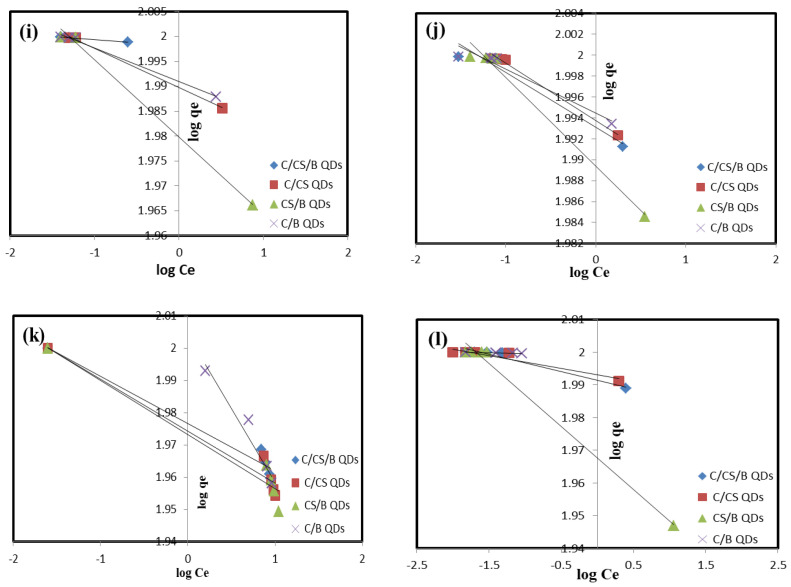
Effect of temperature on the adsorption efficiency of (**a**) Pb(II), (**b**) Cu(II), (**c**) Cr(VI), and (**d**) Mn(II); Langmuir isotherm for (**e**) Pb(II), (**f**) Cu(II), (**g**) Cr(VI), and (**h**) Mn(II); and Freundlich isotherm for (**i**) Pb(II), (**j**) Cu(II), (**k**) Cr(VI), and (**l**) Mn(II).

The effect of temperature on the adsorption capacity of Pb(II), Cu(II), Cr(VI), and Mn(II) on C/CS/B QDs, C/CS QDs, CS/B QDs, and C/B QDs surfaces was investigated from 25 °C to 65 °C at an affixed time ≈ 240 min. As shown in Figure 8, when the temperature increases from 25 °C to 65 °C, the removal of (Pb(II), Cu(II),and Mn(II) by increased, suggesting that the adsorption is an endothermic process due to the enlargement of pore size which in turn increases the rate of diffusion of Pb(II), Cu(II), and Mn(II) across the external boundary layer and in the internal pores. On the other hand, the removal of Cr(VI) by C/CS QDs and CS/B QDs surfaces decreases suggesting that the adsorption is exothermic process due to the decreasing in the boundary layer thickness at high temperatures which in turn facilitates the escape of metal ions away from the adsorbent [28].

In the case of C/CS/B QDs, the removal of Pb(II) increased until 55 °C (i.e., endothermic) then starts to decrease (i.e., exothermic). This may be due to the boundary layer thickness which decreases here after 55 °C. The C/B QDs shows the same behavior after 35 °C (i.e., endothermic before 35 °C and exothermic after that temperature).

All isotherms except C/CS/B QDs for Mn(II) were found to best fit the Langmuir isotherm due to the high value of R^2^ (Table 4). Thus, it can conclude that their surfaces are homogeneous, and the surface adsorption mainly occurs in a monolayer form, while C/CS/B QDs for the Mn(II) surface is heterogeneous in a multi-layer fashion [28].

The negative ΔG values designate a spontaneous sorption process. The ΔS variations exhibited positive values for all adsorbents except C/B QDs for Pb(II) explaining the increased randomness displayed on the adsorbents solution interface during metal ions exchangeable. Conversely, the negative ΔS values of C/B QDs for Pb(II) elucidate the decreased randomness at the C/B QDs solution interface (Table 5) [28].

### 3.3. Application of Quantum Dots as Metal Sensor

**Figure 9:**
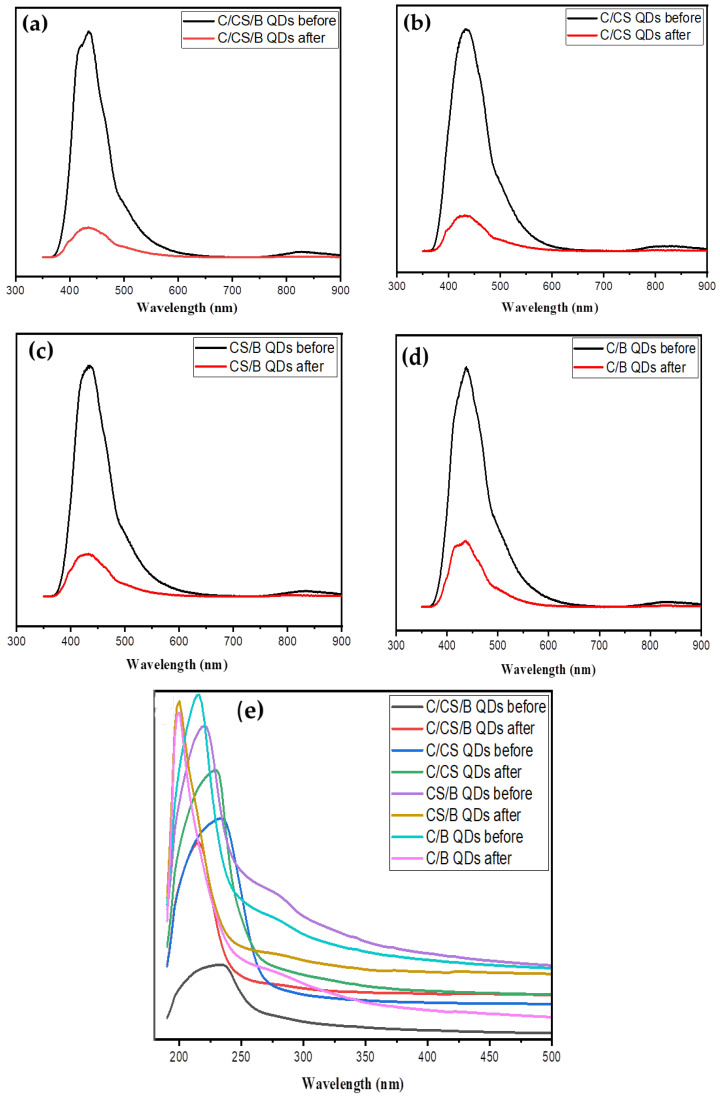
(**a**–**d**) Fluorescent spectra and (**e**) UV spectra of C/CS/B QDs, C/CS QDs, CS/B QDs, and C/B QDs before and after adsorption.

The C/CS/B QDs, C/CS QDs, CS/B QDs, and C/B QDs were excited at 350 nm and showed the maximum emission wavelength at 417.00, 432.00, 435.00, and 438.00 nm, respectively, due to the oxygen vacancy defects of the CQDs’ and GQDs’ surfaces (Figure 9). This difference in the peak position is attributed to the variation in the cross-linking between cellulose, biochar, and chitosan. The emission peaks after the adsorption of metal ions (i.e., Pb(II), Cu(II), Cr(VI), and Mn(II)) were shifted to 434.00, 437.00, 436.00, and 441.00, respectively. The calculated fluorescence quenching efficiency (FQE) was 85.11, 81.59, 79.44, and 70.45%, indicating relatively high sensitivity. The interaction between nitrogenized and oxygenated surface functionalities (–COOH, –OH, and –NH_2_) of cellulose, biochar, and chitosan was responsible for quenching fluorescence efficiency [2]. The reduction in fluorescence intensity after the adsorption of metal ions is due to the fluorescence inner filter effect [2]. Accordingly, the fluorescence quenching mechanism is considered from the fluorescence inner filter effect.

In addition, the high FQE of these findings validated the efficiency of C/CS/B QDs, C/CS QDs, CS/B QDs, and C/B QDs as excellent materials for further utilization in chemical sensing applications.

The UV–vis spectrum of C/CS/B QDs, C/CS QDs, CS/B QDs, and C/B QDs shows typical optical absorption in the UV region. The spectra have intensive peaks at 232.00, 236.00, 222.00, and 218.00 nm due to the π–π* transition of C=C bonds at C/CS/B QDs, C/CS QDs, CS/B QDs, and C/B QDs, respectively [1,3]. A shoulder peak at 284.00, 278.00, and 276.00 for C/CS/B QDs, CS/B QDs, and C/B QDs was assigned to the n–π* transition of C=O bonds, disappearing due to the interaction between C=O of cellulose and NH_2_ of chitosan [3]. The calculated QY was 22.19, 43.80, 38.19, and 39.58% for C/CS/B QDs, C/CS QDs, CS/B QDs, and C/B QDs, respectively. FTIR spectra proved this, which showed a low intensity for the C=O band. The λ_max_ had a slight red shift following the addition of Pb(II), Cu(II), Cr(VI), and Mn(II), indicating the formation of a complex between each of the C/CS/B QDs, C/CS QDs, CS/B QDs, and C/B QDs and metal ions.

### 3.4. Adsorption Comparsion Study

The removal % of the adsorbents for the removal of Pb(II), Cu(II), Cr(VI), and Mn(II) have been compared with those of biochars as adsorbents extracted from other sources reported in the literature. A list showing the removal % of different biochars for the adsorption of Pb(II), Cu(II), Cr(VI), and Mn(II) from aqueous solutions is given in Table 6. As can be seen, the observed removal % of the prepared CQDs for Pb(II), Cu(II), Cr(VI), and Mn(II) are comparable with other low-cost adsorbents.

As chemical sensors: In previous studies, the CQDs prepared from agro-wastes had FQE 49.57% for Cr(VI) [24]. In addition, Yao et al. found that the FEQ % of a mixture of CQDs and QDs was 60%. Accordingly, the prepared CQDs in this study have high FEQ efficiencies, which are 85.11, 81.59, 79.44, and 70.45%, corresponding to C/CS/B QDs, C/CS QDs, CS/B QDs, and C/B QDs, respectively [2,24].

## 4. Conclusions

Finally, we established an eco-friendly, simple, fast method for producing modified quantum dots (QDs) using a microwave from different carbon sources, such as cellulose, chitosan, and biochar. Various sources were selected to study the effect of source on the QDs efficiency. The characterization studies of the prepared QDs found that using a mixture of cellulose and chitosan gave carbon quantum dots (CQDs). In contrast, other combinations showed graphene quantum dots (GQDs), like graphene sheets incorporated with CQDs. The higher correlation coefficient indicated that the adsorption process’s Kinetics could be fitted with the pseudo second-order kinetic model. The prepared quantum dots showed high efficiency toward Cr(II) adsorption following Cu(II), Mn(II), and Pb(II), respectively.

## Figures and Tables

**Table 1:** Raman spectra peak positions, *I*_D_/*I*_G_, and *I*_2D_/*I*_G_ values of C/CS/B QDs, C/CS QDs, CS/B QDs, and C/B QDs.

Parameter	C/CS/B QDs	C/CS QDs	CS/B QDs	C/B QDs
D band (cm^−1^)	–	–	1000	1014
G band (cm^−1^)	1982	1860	1982	1650
2D band (cm^−1^)	2660	2398	2674	2660
I_D_/I_G_	–	–	–	2.02
I_2D_/I_G_	0.37	0.57	0.58	0.63

**Table 2:** TGA/DTG data of; C/CS/B QDs, C/CS QDs, CS/B QDs, and C/B QDs.

Sample	Stage	Temp. (°C)	Max. Temp. (°C)	ML (%)	R^2^	N	A (s^−1^)	ΔH (kj.mol^−1^)	ΔG (kj.mol^−1^)	Δs (kj.mol^−1^)	E_a_ (kj.mol^−1^)	SE
C/CS/B QDs	1st	41.82–264.60	214.05	37.51	0.912	2.0	0.11	66.72	196.75	–0.26	70.76	58 × 10^−1^
	2nd	266.27–357.27	331.02	13.29	0.984	2.0	0.24	14.47	173.06	–0.26	19.49	15 × 10^−2^
		357.40–978.72	397.27	25.86 RW = 23.34%	0.996	2.0	0.24	12.43	188.95	–0.26	18.00 ∑E = 37.49	16 × 10^−2^
C/CS QDs	1st	41.70–276.06	215.76	35.80	0.952	3.0	0.04	133.58	268.25	–0.27	137.64	12 × 10^−1^
	2nd	277.83–361.05	330.05	10.78	0.988	2.0	0.24	14.32	172.55	–0.26	19.34	11 × 10^−2^
		362.71–979.81	400.36	26.59 RW = 26.83%	0.994	2.0	0.24	12.87	190.15	–0.26	18.47 ∑E = 37.81	20 × 10^−2^
CS/B QDs	1st	42.15–270.61	216.53	14.76	0.936	2.0	0.14	57.33	187.05	–0.26	61.40	41 × 10^−1^
	2nd	279.27–379.82	342.99	18.56	0.978	2.0	0.21	32.64	195.13	–0.26	37.76	39 × 10^−2^
		379.90–452.90	403.60	8.18	0.987	2.0	0.23	24.05	202.61	–0.26	29.67	11 × 10^−2^
	3rd	598.23–759.90	663.30	13.16 RW = 58.50%	0.999	3.0	0.20	14.03	264.68	–0.26	21.81 ∑E = 89.24	33 × 10^−3^
C/B QDs	1st	45.26–119.92	96.38	2.55	0.835	2.0	0.007	88.00	187.17	–0.26	91.07	60 × 10^−1^
	2nd	151.98–269.50	217.19	32.58	0.972	2.0	0.17	44.63	173.92	–0.26	48.71	10 × 10^−1^
	3rd	282.05–371.05	340.47	6.18	0.972	2.0	0.24	17.11	178.24	–0.26	22.21	20 × 10^−2^
		372.16–979.23	402.43	9.81 RW = 48.88%	0.994	2.0	0.25	13.10	190.92	–0.26	18.72 ∑E = 89.64	21 × 10^−2^

**Table 3:** Comparison between the estimated adsorption rate constants and correlation coefficients associated with the pseudo first-order, the pseudo second-order rate, and intra-particle diffusion.

Kinetic Model	Metal Ion	Parameter	C/CS/B QDs	C/CS QDs	CS/B QDs	C/B QDs
Pseudo-first order	Pb(II)	q_exp._ (mg/g)	99.75	96.75	92.50	97.25
		q_Calc._ (mg/g)	99.09	93.07	90.24	93.95
		k_1_	35 × 10^−4^	16 × 10^−4^	62 × 10^−6^	74 × 10^−6^
		R^2^	0.722	0.821	0.858	0.769
	Cu(II)	q_exp._ (mg/g)	99.75	98.50	97.25	98.50
		q_Calc._ (mg/g)	94.08	96.18	95.96	97.47
		k_1_	22 × 10^−4^	73 × 10^−6^	26 × 10^−6^	37 × 10^−6^
		R^2^	0.881	0.608	0.845	0.947
	Cr(VI)	q_exp._ (mg/g)	99.97	99.97	99.97	99.75
		q_Calc._ (mg/g)	99.73	99.77	98.61	100.79
		k_1_	78 × 10^−5^	99 × 10^−5^	36 × 10^−4^	23 × 10^−4^
		R^2^	0.904	0.675	0.842	0.865
	Mn(II)	q_exp._ (mg/g)	99.95	98.00	98.00	97.25
		q_Calc._ (mg/g)	96.02	96.62	92.50	96.19
		k_1_	85 × 10^−4^	45 × 10^−4^	31 × 10^−4^	34 × 10^−4^
		R^2^	0.818	0.937	0.920	0.767
Pseudo-second order	Pb(II)	q_Calc._ (mg/g)	5.10	3.44	9.55	3.52
		k_2_	11 × 10^−2^	37 × 10^−1^	14 × 10^−1^	22 × 10^−1^
		R^2^	0.993	0.855	0.994	0.963
	Cu(II)	q_Calc._ (mg/g)	1.91	1.79	3.94	1.33
		k_2_	11 × 10^−2^	94 × 10^−1^	96 × 10^−2^	19 × 10^−1^
		R^2^	0.972	0.824	0.982	0.974
	Cr(VI)	q_Calc._ (mg/g)	0.23	0.031	0.031	0.037
		k_2_	39 × 10^−2^	18 × 10^−1^	11 × 10^−1^	36 × 10^−2^
		R^2^	0.958	0.917	0.973	0.592
	Mn(II)	q_Calc._ (mg/g)	2.39	1.94	7.45	2.11
		k_2_	10 × 10^−1^	43 × 10^−2^	31 × 10^−2^	18 × 10^−1^
		R^2^	0.986	0.931	0.937	0.984
Intra-particle diffusion	Pb(II)	k_p_ (mg·g^−1^·min^−1(0.5)^)	1.53	30 × 10^−1^	25 × 10^−1^	26 × 10^−1^
		C(mg/g)	21.35	8.14	11.46	7.17
		R^2^	0.788	0.834	0.906	0.865
	Cu(II)	k_p_ (mg·g^−1^·min^−1(0.5)^)	8 × 10^−2^	6 × 10^−2^	2 × 10^−2^	1 × 10^−2^
		C(mg/g)	3.23	0.76	4.19	0.196
		R^2^	0.846	0.852	0.954	0.791
	Cr(VI)	k_p_ (mg·g^−1^·min^−1(0.5)^)	23 × 10^−2^	21 × 10^−2^	17 × 10^−2^	29 × 10^−2^
		C(mg/g)	0.39	0.34	2.54	7.66
		R^2^	0.948	0.793	0.559	0.740
	Mn(II)	k_p_ (mg·g^−1^·min^−1(0.5)^)	44 × 10^−1^	35 × 10^−1^	1 × 10^−2^	29 × 10^−1^
		C(mg/g)	8.38	8.84	0.45	7.66
		R^2^	0.537	0.853	0.905	0.811

**Table 4:** Comparison between the estimated adsorption rate constants and correlation coefficients associated with the Langmuir and Freundlich isotherms.

Kinetic Model	Metal Ion	Parameter	C/CS/B QDs	C/CS QDs	CS/B QDs	C/B QDs
Langmuir isotherm	Pb(II)	q_m_ (mg/g)	99.70	96.99	92.45	97.20
		R^2^	0.999	0.999	0.999	0.999
	Cu(II)	q_m_ (mg/g)	97.94	98.16	96.44	98.44
		R^2^	0.999	0.999	0.999	0.999
	Cr(VI)	q_m_ (mg/g)	91.51	90.39	89.81	89.08
		R^2^	0.999	0.999	0.999	0.999
	Mn(II)	q_m_ (mg/g)	97.47	97.97	88.48	99.89
		R^2^	0.448	0.999	0.999	0.999
Freundlich isotherm	Pb(II)	Kf (mg(^1−1/n^) g^−1^ L^1/n^)	7.37	7.31	7.24	7.32
		R^2^	0.994	0.998	0.994	0.994
	Cu(II)	Kf (mg(^1−1/n^) g^−1^ L^1/n^)	7.33	7.34	7.31	7.34
		R^2^	0.959	0.993	0.982	0.947
	Cr(VI)	Kf (mg(^1−1/n^) g^−1^ L^1/n^)	7.21	7.20	7.19	7.42
		R^2^	0.979	0.957	0.978	0.951
	Mn(II)	Kf (mg(^1−1/n^) g^−1^ L^1/n^)	7.32	7.33	7.15	7.38
		R^2^	0.976	0.914	0.991	0.991

**Table 5:** Thermodynamic parameters for C/CS/B QDs, C/CS QDs, CS/B QDs, and C/B QDs adsorption of Pb(II), Cu(II), Cr(VI), and Mn(II).

Kinetic Model		Metal Ion	C/CS/B QDs	C/CS QDs	CS/B QDs	C/B QDs
Δs (kJ/mole)		Pb(II)	83 × 10^−6^	66 × 10^−6^	41 × 10^−6^	−69 × 10^−6^
		Cu(II)	68 × 10^−6^	75 × 10^−6^	65 × 10^−6^	76 × 10^−6^
		Cr(VI)	15 × 10^−4^	15 × 10^−4^	14 × 10^−4^	13 × 10^−4^
		Mn(II)	70 × 10^−5^	73 × 10^−6^	14 × 10^−6^	84 × 10^−6^
ΔH (kJ/mole)		Pb(II)	−50 × 10^−2^	−82 × 10^−1^	−20	−69 × 10^−1^
		Cu(II)	−49 × 10^−1^	−42 × 10^−1^	−89 × 10^−1^	−36 × 10^−1^
		Cr(VI)	22	27	30	18
		Mn(II)	−63 × 10^−1^	−5 × 10^−1^	−32	11 × 10^−1^
ΔG (kJ/mole)	298 K	Pb(II)	−25 × 10^−2^	−25 × 10^−2^	−25 × 10^−2^	−25 × 10^−2^
	308 K		−25 × 10^−2^	−25 × 10^−2^	−25 × 10^−2^	−25 × 10^−2^
	318 K		−26 × 10^−2^	−26 × 10^−2^	−26 × 10^−2^	−26 × 10^−2^
	328 K		−27 × 10^−2^	−27 × 10^−2^	−27 × 10^−2^	−27 × 10^−2^
	338 K		−28 × 10^−2^	−28 × 10^−2^	−28 × 10^−2^	−28 × 10^−2^
	298 K	Cu(II)	−25 × 10^−2^	−25 × 10^−2^	−25 × 10^−2^	−25 × 10^−2^
	308 K		−25 × 10^−2^	−25 × 10^−2^	−25 × 10^−2^	−25 × 10^−2^
	318 K		−26 × 10^−2^	−26 × 10^−2^	−26 × 10^−2^	−26 × 10^−2^
	328 K		−27 × 10^−2^	−27 × 10^−2^	−27 × 10^−2^	−27 × 10^−2^
	338 K		−28 × 10^−2^	−28 × 10^−2^	−28 × 10^−2^	−28 × 10^−2^
	298 K	Cr(VI)	−24 × 10^−2^	−24 × 10^−2^	−24 × 10^−2^	−26 × 10^−2^
	308 K		−27 × 10^−2^	−27 × 10^−2^	−28 × 10^−2^	−26 × 10^−2^
	318 K		−29 × 10^−2^	−29 × 10^−2^	−29 × 10^−2^	−29 × 10^−2^
	328 K		−29 × 10^−2^	−31 × 10^−2^	−30 × 10^−2^	−30 × 10^−2^
	338 K		−31 × 10^−3^	−31 × 10^−3^	−30 × 10^−3^	−30 × 10^−3^
	298 K	Mn(II)	−25 × 10^−3^	−25 × 10^−2^	−28 × 10^−2^	−24 × 10^−2^
	308 K		−25 × 10^−2^	−25 × 10^−2^	−25 × 10^−2^	−25 × 10^−2^
	318 K		−26 × 10^−2^	−26 × 10^−2^	−26 × 10^−2^	−26 × 10^−2^
	328 K		−27 × 10^−2^	−27 × 10^−2^	−27 × 10^−2^	−27 × 10^−2^
	338 K		−28 × 10^−2^	−28 × 10^−2^	−28 × 10^−2^	−28 × 10^−2^

**Table 6:** Removal % of different biochars.

Metals	Source of Biochar	Removal %	References
Pb(II)	bamboo	99.00	[33]
Cu(II)	sunflower	89.40	[34]
Cr(VI)	Walnut Shell	93.00	[35]
Mn(II)	date palm	40.36	[36]

## Data Availability

The datasets used and/or analyzed during the current study are available from the corresponding author upon reasonable request.
